# 

**Published:** 1837-01-01

**Authors:** 


### MEDICO-CHIRURGICAL

### REVIEW,

AND

### JOURNAL

OF

### PRACTICAL MEDICINE.

*(NEW SERIES.)*

VOLUME TWENTY-SIX,

[1st of OCTOBER, 1836, to 31st of MARCH,]

1837.

VOL. VI. of DECENNIAL SERIES.

EDITED

BY JAMES JOHNSON, M.D.

PHYSICIAN EXTRAORDINARY TO THE KING,

AND

HENRY JAMES JOHNSON, Esq.

LECTURER ON ANATOMY AT THE SCHOOL OF ST. GEORGE'S HOSPITAL IN KINNERTON STREET.

Surgeon General's Office
LIBRARY
5-7466
Washington, D.C.

LONDON:

PUBLISHED BY S. HIGHLEY, 32, FLEET STREET,

*Re-printed in New York, by Mr. Wood.*

1837.

W1
ME 15T

PRINTED BY F. HAYDEN,
Little College Street, Westminster.

### BIBLIOGRAPHICAL RECORD;

OR,

*Works received for Review since last Quarter.*

1. A History of British Quadrupeds. By Thomas Bell, FRS. &c. Lecturer on Comparative Anatomy at Guy's Hospital. Part III. price 2*s*. 6*d*. illustrated with Woodcuts and numerous Vignettes. 1st Sept. 1836.

☞ *The account of the Mole is here concluded—the different kinds of Shrew are described—the Badger is included—as is the Otter. The Weasel is commenced. The work is highly deserving of encouragement.*

---

2. Facts and Cases in Obstetric Medicine, with Observations on some of the most important Diseases incidental to Females. By J. T. Ingleby, M.R.C.S. Senior Surgeon to the General Dispensary, &c. Birmingham. One Vol. 8vo, pp. 296. Longman and Co. 1836.

---

3. The Human Brain, its Configuration, Structure, Development, and Physiology, illustrated by Reference to the Nervous System in the Lower Order of Animals. By Samuel Solly, Lecturer on Anatomy and Physiology in St. Thomas's Hospital, &c. Octavo, pp. 402, with 12 Plates. Longman and Co. 1836, price 12*s* 6*d*.

---

4. An Account of the most frequented Watering-places on the Continent, and of the Medicinal Application of their Mineral Springs, with Tables of Analysis, &c. and an Appendix on English Mineral Waters. By Edwin Lee, Esq. M.R.C.S. Octavo, pp. 232. Longman and Co. Oct. 1836.

5. A Medical Vocabulary, or Explanation of all Names, Synonyms, Terms, and Phrases used in Medicine, Surgery, and the relative Branches, with correct Derivation, and Directions for the proper Pronunciation of each, &c. Duodecimo, pp. 185. Carfrae, Edinb. October, 1836, price 4*s.* 6*d.*

☞ *This is really a very clever and compact vocabulary, equally useful to the student and practitioner.*

---

6. A Treatise on Tetanus, being the Essay for which the Jacksonian Prize, for the Year 1834, was awarded by the Royal College of Surgeons in London. By Thomas Blizard Curling, Assistant-Surgeon to the London Hospital, and Lecturer on Morbid Anatomy. Royal 8vo. pp. 236. Oct. 1836, price 8*s.* Rivington—Highley.

---

7. Guy's Hospital Reports, No. 3, Sept. 1836. Edited by G. H. Barlow, M.D. and J. P. Babington, M.A. Octavo. Highley, Oct. 1st, 1836.

---

8. The Clinique Medicale; or Reports of Medical Cases. By G. Andral, M.D. Condensed and translated by D. Spillan, M.D. Part V. containing Diseases of the Abdomen. Renshaw, Oct. 1836.

---

9. A History of British Quadrupeds. By Thos. Bell, F.R.S. Part IV. price 2*s.* 6*d.*

☞ *Weasels concluded—Marten's Wild Cat—Domestic Cat. All very masterly.*

---

10. On the Disease of the Hip-joint: With plain and coloured Plates. By William Coulson, Consulting-Surgeon to the London Lying-in Hospital—late Surgeon to the General Dispensary, &c. Quarto, pp. 111, with many Plates. Oct. 1836. Thomas Hurst.

---

11. Statistics of Phrenology; being a Sketch of the Progress and present State of that Science in the British Isles. By Henry C. Watson. Longman and Co. 1836.

---

12. A History of British Quadrupeds, &c. Part V.

☞ *This Part is occupied with the "Dog," and is executed with great ability.*

---

13. A System of Phrenology. By Geo. Combe In two Vols. 8vo, 4th Edition. Edinb. and London, Nov. 1836.

☞ *Large additions have been made to the present edition—improved cuts have been used—and reference has been made to various other phrenological works, so as to render this Edition an index to the general literature of the science.*

---

14. The British Medical Almanack for 1837. Edited by William Farr. Octavo, pp. 215. Sherwood and Co. 1836, price 3s. with Supplement.

☞ *This Work is more copious than that of Mr. Foote. It is a pity that there should be two works of the same kind. One must hurt the other; and the profession cannot afford to buy both.*

---

15. Medical Report of the Managers of the Lunatic Asylum of Aberdeen. May 26th, 1836.

---

16. Outlines of a Course of Lectures on Medical Jurisprudence. By Thos. Stewart Trail, M.D. Regius Professor of Medical Jurisprudence in the University of Edinburgh. 1836.

---

17. Elements of the Practice of Physic, presenting a View of the present State of Special Pathology and Therapeutics. By David Craigie, M.D. &c. Vol. I. pp. 942. A. and C. Black, Edinb. Nov. 1836.

☞ *This volume embraces fevers, cutaneous inflammations, and mucous inflammations.*

---

18. Catalogue Raisonnée, or Classified Arrangement of the Books in the Library of the Medical Society of Edinb. Octavo, pp. 342. Edinb. Nov. 1836.

---

19. An Examination of Mr. Scott's Attack upon Mr. Combe's Constitution of Man. By Hewett C. Watson. Octavo, price 6*d.* Edinb. 1836.

---

20. A practical Treatise on the Management and Diseases of Children. By Richard T. Evanson, M.D. Professor of Medicine, and Henry Maunsell, M.D. Prof. of Midwifery, in the Royal College of Surgeons, Ireland. Small 8vo, pp. 527, price 7*s.* 6*d.* Dublin, Nov. 1836.

---

21. Statement of the Proceedings which have led to the Resignation of the Medical Officers of the Northern Dispensary. Octavo, sewed.

☞ *As far as we can see, the medical officers abovementioned had good reason for resigning, and deserve praise for their spirited and unanimous conduct.*

22. The Principles of Surgery. By Jas. Syme, F.R.S. E. Professor of Clinical Surgery in the University of Edinburgh. Second Edit. Dec. 1836.

☞ *Greatly enlarged in matter, but, by means of small type, not increased in size.*

---

23. Lectures on the Morbid Anatomy of the Mucous and Serous Membranes. In two Vols. By Thomas Hodgkin, M.D. Vol. I.—the Serous Membranes. November, 1836.

---

24. The Medical Pocket-book for 1837. By John Foote, jun. Renshaw and Rush.

*A very useful vade-mecum.*

---

25. A familiar Treatise on the Natural History of the Silk-worm. By H. W. Dewhurst, Esq. &c. Duodecimo, with a Plate, price 2*s* 6*d*.

---

26. Engravings of Varieties in the Origin, Course, and Distribution of the Arteries, chiefly from Original Observations and Drawings, with descriptive Letter-press. By John Knox, F.R.S. E. *Fasciculus* 1. *External Carotids—Radial Artery—Peroneal Artery—Anterior Tibial Artery.* Edin. Nov. 1836.

---

27. A Bedside Manual of Physical Diagnoses, applied to Diseases of the Lungs, Pleura, Heart, &c. By Charles Cowan, M.D. Translator of Louis on Phthisis. 18mo, pp. 58. Sherwood, 2*s*. 6*d*.

☞ *This is the most concentrated and correct exposition that can be found.*

---

28. Phrenology vindicated; being a Reply to an Article in the Quarterly Review for Sept. 1836. By Jos. Toulman Smith, of Lincoln's-Inn, Esq. Octavo, pp. 80, sewed, Longmans, 1836.

---

29. A Series of Anatomical Plates, &c. By Jonas Quain and Wm. J. E. Wilson. Fasciculi 30 to 42 inclusive, price 2*s*. each.

---

30. Animal Magnetism and Homœopathy. By E. Lee. Octavo, sewed.

---

31. A Treatise on Consumption, &c. By William Sweelser, M.D. Boston, 1836.

---

32. A Practical View of Homœopathy; being an Address to British Practitioners on the general Applicability and superior Efficacy of the Homœopathic Method in the Treatment of Disease; with Cases. By Stephen Simpson, M.D. Octavo. Baillière, Nov. 1836.

---

33. Experimental Researches into the Physiology of the Human Voice. A Memoir, by John Bishop, M.R.C.S. &c. Octavo, pp. 24. with Plates.

---

35. Elements of Medicine. Vol. I. on Morbid Poisons. By Robert Williams, M.D. Senior Physician to St. Thomas's Hospital. Octavo, pp. 342.

☞ *This valuable volume treats of Typhus, Scarlatina, Measles, Variola, Varicella, Erysipelas, and Pertussis. It will be reviewed in our next Number.*

---

36. A History of British Quadrupeds. By Thomas Bell, F.R.S. Part VI. price 2s. 6d.

☞ *Irish Greyhound, or Wolf-dog—Newfoundland Dog—Bull Dog—Mastiff—Vulpes—Phoca, Seal—Sea-calf—Harp seal—Great Seal—Grey Seal—Sea-horse. All well described and admirably delineated.*

---

36. Observations on the Construction and Use of the Respirator; an Instrument for facilitating the Respiration of Persons suffering under Pulmonary and Bronchial Affections, by warming the Air inspired, &c. Octavo, pp. 12. Hodson, London, Dec. 1836.

---

37. Anatomical, Pathological, and Therapeutical Researches upon the Disease known under the Name of Gastro-Enterite, putrid, adynamic, ataxic, or typhoid Fever, &c. compared with the most common Acute Diseases. By P. Ch. A. Louis, M.D. &c. translated from the original French. By Henry Bowditch, M.D. In two Vols. 8vo. Boston (America) 1836, and Kennet, York-street, Covent-garden.

☞ *This is a highly valuable work, and ought to be in the hands of every practitioner in this country.*

---

38. Di una Legatura dell'Arteria Ascellare all'Uscire di Sotto alla clavicola per Emorragia al Cavo Ascella. Osservatione seguita da Pratiche considerazioni sulli Emorragie Arteriose, e sul Processo Operativo da preferirsi nella suddetta Operazione. Di Natale Catanoso, M.D. &c. Di Messina.

☞ *In our next.*

